# Transcriptional profiling of stellate ganglia from normotensive and spontaneously hypertensive rat strains

**DOI:** 10.1038/sdata.2018.123

**Published:** 2018-06-26

**Authors:** Harvey Davis, Emma N. Bardsley, David J. Paterson

**Affiliations:** 1 Burdon Sanderson Cardiac centre, Department of Physiology, Anatomy and Genetics, University of Oxford, Parks Road, OX1 3PT, UK; 2 OXION initiative, Department of Physiology, Anatomy and Genetics, University of Oxford, Parks Road, OX1 3PT, UK

**Keywords:** Hypertension, Gene expression, Autonomic nervous system, Transcriptomics

## Abstract

The course of hypertension remains poorly understood, although impairment of the sympathetic nervous systems is thought to play a role in its aetiology. In this study, RNA-sequencing (RNAseq) was used to identify transcriptomal differences in the sympathetic stellate ganglia between 16-week-old normotensive Wistar rats and spontaneously hypertensive rats (SHR). Sequencing quality was assessed by FastQC and quasi-mapping rate by Salmon. Differential expression results were confirmed by real time reverse transcriptase Quantitative Polymerase Chain Reaction (*q*RT-PCR). RNAseq analysis was found to be predictive and representative of transcriptomal changes when compared to *q*RT-PCR by correlation analysis. Whether these changes underpin physiological sympathetic phenotypes associated with hypertension remains to be established, however this dataset identifies lead transcripts as *a priori* targets for further investigation.

## Background & Summary

Hypertension represents one of the biggest killers in the developed world, yet its underlying aetiology is poorly understood^1^. Previous studies have highlighted molecular and functional alterations in the cardiac-innervating sympathetic stellate ganglia as one component that might predict and contribute to the development of the disease^2–4^. Here RNA-sequencing (RNAseq) was used to provide a hypothesis neutral and unbiased exploration of gene expression changes in the stellate ganglia in hypertension as a prelude to the identification of novel molecular targets for further investigation.

The spontaneously hypertensive rat (SHR) is one of the most commonly studied experimental models of idiopathic hypertension^5,6^, with a disease progression mirroring that of the typical clinical phenotype^7^. The development of a hypertensive phenotype occurs from age 5-6 weeks in the SHR^8–10^ and is well established by the 16-week time point^9,11^. This model is derived from the same Wistar colony as the inbred Wistar-Kyoto (WKY) normotensive strain^8^, with both Wistar and WKY deemed relevant controls^12,13^. Wistar rats were chosen as normotensive controls due to the divergent genetic heterogeneity of the WKY strain^14,15^. The phenotype of the SHR bears similarities to other rodent based models of hypertension, showing comparable levels of cardiac hypertrophy, and endothelial and renal dysfunction^5^.

Whole stellate ganglia were used for RNAseq with the assumption that neurons represented a primary cell population^16,17^. This approach allowed for a snapshot of the cells in a more physiological state, as previous studies in the related superior cervical ganglia have shown that the electrophysiological properties of intact and dissociated neurons differ, which suggests a possible change in neuronal phenotype^18^. Further to this, dissociation of neurons from the stellate ganglia may introduce an unknown bias for alternative cell subpopulations due to the relatively harsh dissociation process involving trypsin and collagenase^3^.

Results obtained from differential expression analysis were confirmed by *q*RT-PCR. The direction and magnitude of these changes were found to be comparable between these two alternate methods. Quality control assessment of the data and the differential expression analysis also revealed the sequencing to be accurate. Together these results suggest that our RNAseq data are predictive of gene expression changes in the stellate ganglia in hypertension, and that the data enables further molecular characterisation.

## Methods

The methods described below are an expansion of the methods outlined in our related work^19^.

### Animals

All experiments were performed in accordance with the UK Home Office Animal Scientific Procedures Act 1986 (ASPA) and approved by the University of Oxford (PPL 30/3131; David J. Paterson).

### Stellate ganglia isolation

Right stellate ganglia were removed from four biological replicates per Wistar and SHR strains at 16-weeks of age (Table 1). Rats (Enivgo, UK) were euthanised by overdose of pentobarbitol whilst under 5% isoflurane and exsanguinated. The stellate ganglia were isolated and placed into ice cold Ca^2+^ and Mg^2+^ free Hanks salt solution (ThermoFisher, MA, US). The ganglia were carefully desheathed and cleaned in preparation for immediate RNA extraction.

### RNA extraction

The clean stellate ganglia were chopped, triturated and digested in RNeasy RLT buffer and RNA was extracted using the standard RNeasy Micro kit protocol (Qiagen, CA, US). Following extraction, sample quality was confirmed using a Bioanalyzer 2100 (Agilent Technologies, CA, US). All replicates had RNA integrity values >8.5. The quantity of RNA was assessed at this stage using a Qubit 2.0 fluorometer and a Qubit high sensitivity RNA assay kit (Invitrogen, CA, US).

### RNA Sequencing

RNA samples were processed by the high-throughput Genomics group at the Wellcome Trust Centre for Human Genetics. Samples were amplified by SMARTer (ClonTech, CA, US) due to the low quantities of RNA extracted from individual stellate ganglia. Library preparation used total RNA with the Illumina TruSeq Stranded mRNA Library Prep Kit (Illumina, CA, USA). Samples were sequenced using an Illumina Hi-Seq 4000 (Illumina, CA, USA), with samples repeated across two lanes to reduce technical error and increase read depth. The samples were sequenced with a paired end protocol (75 base pairs).

### Quasi-mapping

The quasi-mapping function of Salmon (Version 0.8.2) was used with GC bias correction and positional bias correction enabled^20^. For each sample both paired ends for both lanes were run together, effectively combining the multiple files per sample into one output. The index for mapping was built from the UCSC refseq transcriptome for rn6 as obtained from the http repository of UCSC (http://hgdownload.soe.ucsc.edu/goldenPath/rn6/bigZips/). Data demonstrating the validity of this method are shown in Table 2.

### Differential expression analysis

**Figure 1:**
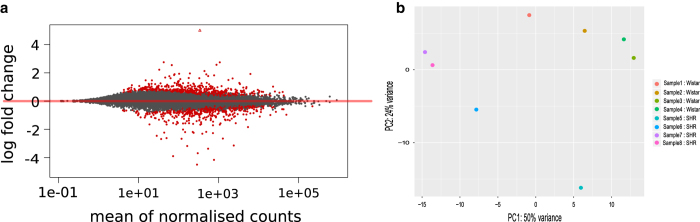
Assessment of differential expression analysis. (**a**) MAplot of DESeq2 (Version 1.16.1) output indicating genes which are significantly differentially expressed (red circles), non-significantly differentially expressed (grey circles), and significantly expressed outside of the 4 to -4 log fold change limit (red triangles). (**b**) PCA plot of DESeq2 (Version 1.16.1) output assesses differences within the dataset via linear principal components.

The output of Salmon was imported into the R environment using the Rstudio software package (R Version 3.4.2; R studio version 1.1.383). This was performed via Tximport (Version 1.4.0)^21^, which converted data from transcript level to gene level during the importing process. Differential expression analysis was performed using DESeq2 (Version 1.16.1)^22^ using the automated DESeq2 function. After differential analysis, DESeq2's LFCShrink function was applied to shrink Log_2_ fold changes. Analysis was subsequently summarised in an MAPlot^22,23^ (Fig. 1a), in which the significance of differential expression results is compared with fold change values and the mean number of normalised counts for a gene. For genes which were significantly differentially expressed between strains the dots appear red, and non-significantly differentially expressed genes appear grey. This significance value is based upon a Wald-significance test (adjusted *P*<0.1)^22^. The MA plot shows the expected results; most genes were not significantly different and the threshold for significance decreases with mean expression value. This is in accordance with DESeq2's analysis method^22^. Further confirmation of the analysis method was achieved via a principal component analysis (PCA) plot^22^ (Fig. 1b). This simplifies the differences between samples into principal components on which differences between samples can be more easily viewed. Generally, there was a separation between the SHR and Wistar samples, suggesting a unifying transcriptomal trend amongst the biological replicates from each strain.

## Data Records

Raw fastQ files produced by RNA-sequencing are deposited in NCBI Sequence Read Archive (Data Citation 1) and Gene Expression Omnibus (Data Citation 2). Key outputs of the analysis were deposited in Gene Expression Omnibus (Data Citation 2).

## Technical Validation

**Figure 2:**
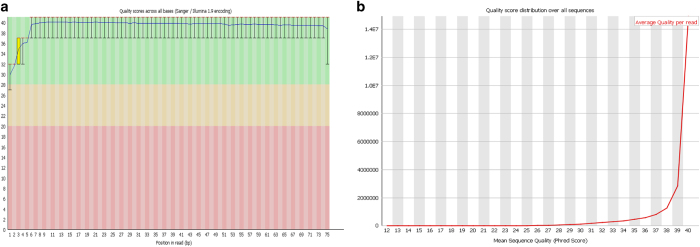
Summary of FASTQC derived base quality scores for first sample in dataset, Wistar 1 mate 1 lane 1. (**a**) Summary of quality scores across all bases relative to the quality threshold of 30. (**b**) Mean quality score of all sequences (Phred score) relative to a chosen threshold of 30, indicating a 99.9% chance that a base is called correctly.

**Figure 3:**
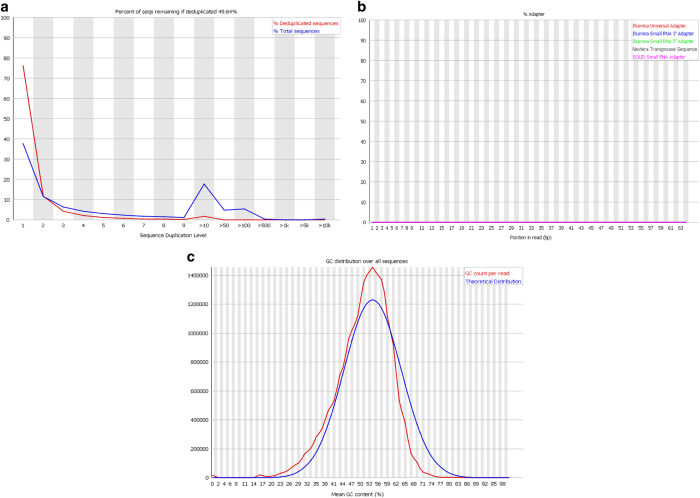
Summary of FASTQC output for first sample in dataset, Wistar 1 mate 1 lane 1. (**a**) Comparison of deduplicated sequences and total sequences reveals the frequency of repeatedly read sequences. (**b**) An assessment of the adapter contamination of the sample. (**c**) Assessment of sample GC bias; observed GC base content per read (red) compared to an expected model (blue).

FastQC High Throughput Sequence QC Report (Version 0.11.7)^24^ was used to assess the quality of the raw Fastq files. The standard of the sequencing quality is represented in Figs. 2 and 3, which show quality control data from Wistar 1 mate 1 lane 1. The quality across all bases is demonstrated in Fig. 2a, showing that most bases had a quality score greater than 30, representing a greater than 99.9% probability that the base has been correctly called in ref. 25. This is reinforced by Fig. 2b which showed a range of mean sequence quality scores that were also typically higher than 30. Figure 3a, highlights sequence duplication, the erroneous repeated reading of a sequence. More sequences were duplicated than expected, but this is possibly an artefact of the SMARTer amplification process. There was no detectable adapter contamination in the sample (Fig. 3b). Finally, Fig. 3c shows that there was a slight Guanine-Cytosine (GC) bias to the data, however this can be accounted for in the alignment/mapping stage. These trends are supported throughout the dataset and indicate the general high quality of the raw sequencing data.

Differential expression analysis was supported by real time reverse transcriptase Quantitative Polymerase Chain Reaction (*q*RT-PCR) experiments on biological replicates from the left stellate ganglia of the sample animals used for RNAseq. RNA was extracted in the same manner as described for the RNA-sequencing experiments. RNA was subsequently converted to cDNA using a Superscript III VILO kit (ThermoFisher, MA, US). Sample quality was confirmed after cDNA preparation using a NanoDrop Lite to calculate the 260/280 ratios. Samples with a ratio of <1.7 or >1.85 were discarded. *q*RT-PCR was then performed using the following TaqMan probes: *Chrnb4* (Rn00583822_m1); *Gabra1* (Rn00788315_M1); *Gabra2* (Rn01413643_m1); *Glrb* (Rn00583966_M1); *Gria1* (Rn00709588_m1); Gria2 (Rn00568514_M1); *Gria3* (Rn00583547_m1); *Grik1* (Rn01458414_M1); *Grik2* (Rn00570853_m1); *Grin2b* (Rn00680474_M1); *Htr3a* (Rn00667026_m1); *Htr3b* (Rn00573408_M1); *P2rx3* (Rn00579301_m1); *P2rx4* (Rn00580949_m1); *P2rx6* (Rn00562354_m1) (ThermoFisher, MA, US). Samples were normalised against beta-2-microglobulin (*B2m*; Rn00560865_m1) Glyceraldehyde 3-phosphate dehydrogenase (Gapdh; Rn99999916_s1). Both control genes were found to be stably expressed between strains by DESeq2 differential expression analysis (B2m, *P*=0.286; Gapdh, *P*=0.972). Experiments were performed as in the manufacturer’s protocol, using an ABI Prism system (ThermoFisher, MA, US).

**Figure 4:**
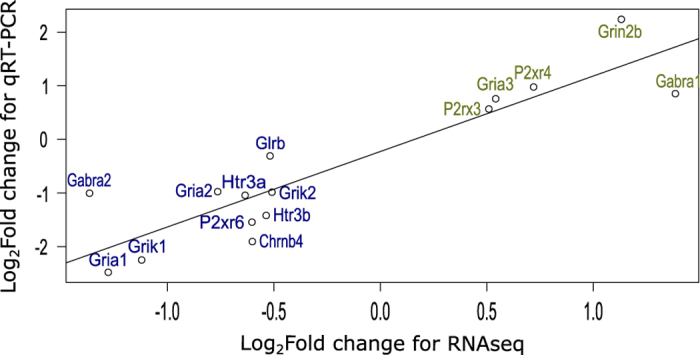
Correlation analysis comparing qRT-PCR and RNAseq analysis of differential expression between Wistar and SHR strains. Correlation analysis comparing genes determined to be significantly different by RNAseq analysis against qRT-PCR technical validation normalised to B2m (r=0.8998, *P*=4.92e-06). Line of fit based on Pearson’s Correlation Coefficient. Green text indicates an increase in expression and blue text indicates a decrease in expression.

Fold changes were calculated by the ΔΔCt method^26^, averaged between technical and subsequently biological replicates to achieve an average fold difference. We then assessed the correlation between the fold changes achieved by *q*RT-PCR and RNAseq to assess the validity of our analysis^27^. Correlation analysis revealed a strong correlation (Fig. 4) (r=0.899, *P*=4.92e-06) between the two methods, suggesting our RNAseq dataset and analysis are highly predictive.

### Code availability

Information on any choices or variables chosen and package version numbers are stated within the manuscript.

## Additional information

**How to cite this article**: Davis, H. *et al*. Transcriptional profiling of stellate ganglia from normotensive and spontaneously hypertensive rat strains. *Sci. Data* 5:180123 doi: 10.1038/sdata.2018.123 (2018).

**Publisher’s note**: Springer Nature remains neutral with regard to jurisdictional claims in published maps and institutional affiliations.

## Supplementary Material



## Figures and Tables

**Table 1:** Overview of experimental design.

Subjects	Protocol 1	Protocol 2	Protocol 3	Data	
Wistar1	Right stellate ganglia dissection	RNA extraction	RNAseq	GSE110197	SRP132271
Wistar2	Right stellate ganglia dissection	RNA extraction	RNAseq	GSE110197	SRP132271
Wistar 3	Right stellate ganglia dissection	RNA extraction	RNAseq	GSE110197	SRP132271
Wistar4	Right stellate ganglia dissection	RNA extraction	RNAseq	GSE110197	SRP132271
SHR1	Right stellate ganglia dissection	RNA extraction	RNAseq	GSE110197	SRP132271
SHR2	Right stellate ganglia dissection	RNA extraction	RNAseq	GSE110197	SRP132271
SHR3	Right stellate ganglia dissection	RNA extraction	RNAseq	GSE110197	SRP132271
SHR4	Right stellate ganglia dissection	RNA extraction	RNAseq	GSE110197	SRP132271
Protocols are indicated for each biological replicate per condition, with associated data repository.					

**Table 2:** Summary of mapping rate by Salmon.

Subjects	Mapping rate	Number of mapped reads	Data
Wistar 1	78.66%	35509076	GSE110197
Wistar 2	75.57%	30112270	GSE110197
Wistar 3	78.38%	34634784	GSE110197
Wistar 4	75.18%	33764576	GSE110197
SHR 1	70.63%	31194868	GSE110197
SHR 2	78.47%	25820298	GSE110197
SHR 3	68.50%	32573670	GSE110197
SHR 4	72.60%	34026720	GSE110197
Number of mapped reads refers to output of Salmon (version 0.8.2) with associated repository information.			
